# Towards Non-Invasive Intravital Microscopy: Advantages of Using the Ear Lobe Instead of the Cremaster Muscle

**DOI:** 10.3390/life13040887

**Published:** 2023-03-27

**Authors:** Iara Mota-Silva, Miguel A. R. B. Castanho, Ana Santos Silva-Herdade

**Affiliations:** Instituto de Medicina Molecular João Lobo Antunes, Faculdade de Medicina, Universidade de Lisboa, 1649-004 Lisboa, Portugal

**Keywords:** intravital microscopy, leukostasis, inflammation, microcirculation

## Abstract

Inflammation is essential in the protection of the organism and wound repair, but in cases of chronic inflammation can also cause microvasculature deterioration. Thus, inflammation monitorization studies are important to test potential therapeutics. The intravital microscopy (IVM) technique monitors leukocyte trafficking in vivo, being a commonly used procedure to report systemic conditions. Although the cremaster muscle, an established protocol for IVM, may affect the hemodynamics because of its surgical preparation, only male animals are used, and longitudinal studies over time are not feasible. Thinking how this impacts future studies, our aim is to understand if the IVM technique can be successfully performed using the ear lobe instead of the cremaster muscle. Elevated IL-1β plasmatic concentrations confirmed the systemic inflammation developed in a diabetic animal model, while the elevated number of adherent and rolling leukocytes in the ear lobe allowed for the same conclusion. Thus, this study demonstrates that albeit its thickness, the ear lobe protocol for IVM is efficient, non-invasive, more reliable, cost-effective and timesaving.

## 1. Introduction

Inflammation plays a vital role in the protection of the human organism against the action of invasive pathogens; it is also beneficial in tissue and wound repair in acute and transient cases [1]. However, in the context of chronic inflammation, as is the case of systemic inflammation caused by diabetes mellitus (DM), it may lead to undesirable effects caused by the deterioration of the microvasculature [1,2]. DM is majorly characterized by high blood glucose levels, known as hyperglycemia [3]. This DM’s hallmark is responsible for the deregulation of several metabolic pathways, which ultimately contribute to increased sensitivity to oxidative stress, affecting pro-inflammatory transcription factors, such as nuclear factor-kappa B (NF-kB) [4,5]. Consequently, systemic inflammation is induced through the expression and activation of pro-inflammatory cytokines and adhesion molecules [6]. Under inflammatory conditions, the endothelium presents increased adhesive properties, particularly towards leukocytes. This happens because the cytokines induce the secretion of E- and P-selectins on the cells of the vascular walls and L-selectins on the leukocytes [7,8,9,10]. Thus, mediated by the interaction between selectins and their ligands, rolling on the luminal surface of vessels is the first step in leukocyte inflammatory dynamics [7,9,10]. Afterwards, the cytokines present on the vessels may promote conformational changes and activation of the integrins present on the rolling leukocytes, resulting in their firm adhesion to the endothelial cells. The last step of the leukocyte inflammatory dynamics is the transendothelial migration, from the vessel’s lumen to the tissue [7,9,10]. Overall, in cases of chronic inflammation, this cascade of events results in the elevated capture and adherence of leukocytes to the blood vessel walls, resulting in leukostasis or hyperleukocytosis. Leukostasis in the blood vessels affects the blood flow, decreasing tissue perfusion and increasing leukocyte migration into the tissues [11,12,13,14]. The increase in leukocyte transmigration through the endothelial walls results in the loss of integrity of these structures [11,12,13,14,15]. As the endothelial walls lose their integrity, the microvasculature deteriorates, and the histological architecture of many tissues of the organism may be affected [11,12,13,14,15]. Microcirculation provides steady blood flow to tissues and organs, despite changing metabolic conditions, and thereby contributes to the maintenance of homeostasis [16,17]. Thus, the assessment of the state of inflammation in studies in vivo is necessary to test the efficacy of potential therapeutics.

Intravital microscopy (IVM) is a well-known technique to monitor leukostasis and the anti-inflammatory action of drugs as it can reliably assess the stage and severity of the inflammation directly in tissues that report the global status of the organism [2,18,19]. Specifically, it allows for the direct visualization and study of biological processes, such as the leukocyte trafficking in vivo [8]. Worthy of note, this technique enables real-time quantitative recordings and their computational analysis [2]. Understandably, it became a valuable tool transversal to fields such as neurobiology, immunology, medicinal chemistry, and tumor biology. The cremaster muscle is widely used in studies with IVM experiments for enabling transillumination and confocal microscopy due to its thinness, which is also important in the study of topically applied molecules [20,21]. However, the preparation procedures of the cremaster for this technique may affect the muscle hemodynamics [21]. This may be explained by the unintentional and imminent local trauma caused by the surgery to expose the muscle, which might result in slower blood flow and an excessive number of adherent leukocytes to the endothelium in some preparations [22]. Importantly, another very straining limitation is that only male mice can be used when the cremaster is used, which abrogates attempts to study gender-correlated effects and raises issues on the ethical use of animals as female mice are discarded. Nevertheless, other organs have already been used in IVM for immunological studies, such as the intestine or the brain [23], and in those tissues, the use of males is not a limitation, but there are other limitations than need to be considered. The mesentery, in general, is a delicate tissue that requires great care when handled because the activation of the resident mast cells can release histamine and then induce leukocyte rolling. For this reason, the murine mesentery should not be exteriorized for more than 30 min without becoming activated, thus limiting observation time points.

The brain was considered for a long time to be an organ difficult to handle because the visualization of cellular interactions within the brain always needs a surgical procedure. Different approaches can be performed, but the thickness of the tissues means that cellular activity within this vascular bed must be observed via fluorescence. However, labelling by dyes often requires cannulation, which has its own risks.

However, all of those models are invasive procedures that cannot be used in longitudinal studies over time. For instance, it is impossible to follow experimental drug treatments at regular time points over large intervals. In an attempt to overcome the setbacks of cremaster-based methodologies, our aim was to study the advantages of using ear-lobe-based IVM methodologies, regarding the assessment of systemic inflammation status. The rolling and adherent leukocytes can be observed in the post-capillary venules (PCVs) using the IVM technique [18,24]. In a previous study [2], we found evidence through IVM, suggesting that changes in the microcirculation, caused by chronic inflammation (mice developed DM for 5 months), develop in parallel in the cremaster and in the ear lobe. However, in this particular study, the assessment of the microcirculation in the ear lobe using intravital microscopy was reported to be very difficult and almost impossible due to the thickness of the tissue. Considering this, we were prompted to determine the conditions in which the ear lobe can replace the cremaster muscle in IVM and carried out a thorough and systematic comparative study to validate ear-lobe-based indicators as reliable markers of chronic inflammation. Briefly, we compared experimental indicators of inflammation by IVM in the cremaster muscle and in the ear lobe while assessing the state of systemic inflammation by the levels of IL-1β cytokine in a diabetic animal model. This pro-inflammatory cytokine plays an important role in chronic diseases, which is the status we aim to address in this study [25,26]. Although the ear lobe (265 μm ± 15.1 μm) is much thicker than the cremaster (98.5 μm ± 2.0 μm), its preparation procedure is much simpler and faster, and it is not invasive [1,2,3].

## 2. Materials and Methods

### 2.1. Ethics Statement

The animal’s welfare was in accordance with the Directive of the European Community 2010/63/EU that mentions the protection of animals used for economic and other scientific ends, as well as with the Portuguese Legislation Law 113/2013. The experiments performed on the animals were approved and carried out according to the guidelines of the ethical committee of the Animal Welfare Body of Instituto de Medicina Molecular (ORBEA-IMM) and were submitted to Direção Geral de Alimentação e Veterinária (DGAV), obtaining the legal authorization.

### 2.2. Animals and Housing

The methods of this research are described according to the ARRIVE guidelines. The animals used were 5-weeks old male Lys-EGFP-ki C57BL/6 mice [27]. These mice have the enhanced Green Fluorescent Protein (EGFP) knock-in mutation in the lysozyme M (Lys) locus, which enabled the visualization of green fluorescent neutrophils. The animals were given water ad libitum. The housing was organized in groups with a maximum of 5 animals per cage, at a temperature of 22 to 24 °C and humidity between 45 and 65% in a controlled room, with a 14 h light/10 h dark cycle. As a randomization strategy, the cages of the 24 animals included in this study were kept in the same rack and in the same row, and the experimental groups were randomly distributed by the cages.

To chemically induce diabetes, 7 male mice were administered, via intraperitoneal injection, with a single dose (140 mg/kg) of Streptozotocin 14 mg/mL in citrate buffer 0.1 M, pH 4.5, after 5 h fasting (100 μL/10 g BW) [2]. The weight and blood glucose levels of diabetic (STZ-injected) mice were measured 48 h after the injection and then at least once a week for 4 months. The mice were considered diabetic when the fasting glycemia values were higher than 250 mg/dL for at least 3 consecutive measurements. The control group, constituting 10 non-diabetic mice, was not injected with STZ and was monitored in the same time points of the diabetic mice. To measure the blood glucose, a needle (23 G) was used to do a tail vein prick and allow a drop of blood to be collected in the glucose test strips connected to the glucometer (Sensolite Nova Plus, Hungary). In order to maintain the blood glucose levels <600 mg/dL, insulin was subcutaneously injected, as needed. Besides weight and blood glucose levels, all the mice’s general appearance and food/water ingestion was evaluated during the monitorization period. Mice were humanely euthanized, and excluded from this work, in case of: severe ulceration, general debilitated state, and/or lost more than 20% of their body weight. If the mice were not considered diabetic after the injection with STZ, they were also excluded from the study. The number of animals used in this study was estimated based on previous studies [2].

The intravital microscopy procedures were performed in the mice’s ear lobe and cremaster, 4 months after the onset of diabetes in STZ-treated animals and 4 months after the beginning of the experiment in control mice. After IVM, blood was collected from the animals to heparinized tubes, using the cardiac puncture technique. Finally, the animals were euthanized.

### 2.3. Study Design

In this study, animals were divided into two groups, control or nondiabetic (*n* = 10) and diabetic (*n* = 7) animals. The investigator was unable to be blinded to the mouse group due to the blood glucose levels of the mice, and because the study was performed by one investigator. In each group, the number of rolling and adherent leukocytes in the post-capillary venules was quantified, as well as the rolling velocity. For this, the IVM technique was used to visualize and record microcirculation in the cremaster muscle and ear lobe. Additionally, the concentration of IL-1β in plasma was determined by ELISA in both the experimental groups.

### 2.4. Intravital Microscopy

The mice were anesthetized with a cocktail constituted by 100 mg/mL of ketamine and 20 mg/mL of xylazine, via an i.p. injection (100 μL/10 g BW). During the experiment and data acquisition, the room temperature was 22 °C, and the animals were always placed over thermostatically controlled instruments at 37 °C. The anesthesia depth of the mice was continuously assessed during the experiment by checking the whiskers of the mouse for any movement and by pinching the toe. A heating pad was continuously kept below the mice and mice cage before and after the anesthesia. The cremaster and ear lobe preparation was conducted on a support with a transparent lamella, placed above the microscope heated stage, through which the trans-illuminated tissue was visualized. The intravital microscopy procedure was performed in the ear lobe and subsequently in the cremaster.

**Figure 1:**
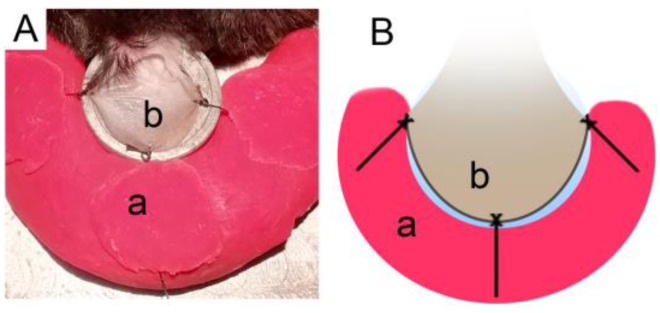
Ear lobe preparation. Photographed (**A**) and illustrated (**B**) representations of ear lobe preparation procedure. (a) Plasticine; (b) ear lobe.

For the preparation of the ear lobe, the fur behind the analyzed ear had to be shaved using a body groomer machine, and no products. Afterwards, the mice’s ear was placed over the lamella of the heated support, as explained previously, and held by plasticine using 3 stiches made of silk suture (B.Braun; Black Silk 5/0 HR20 1/2 45CM) (Figure 1).

**Figure 2:**
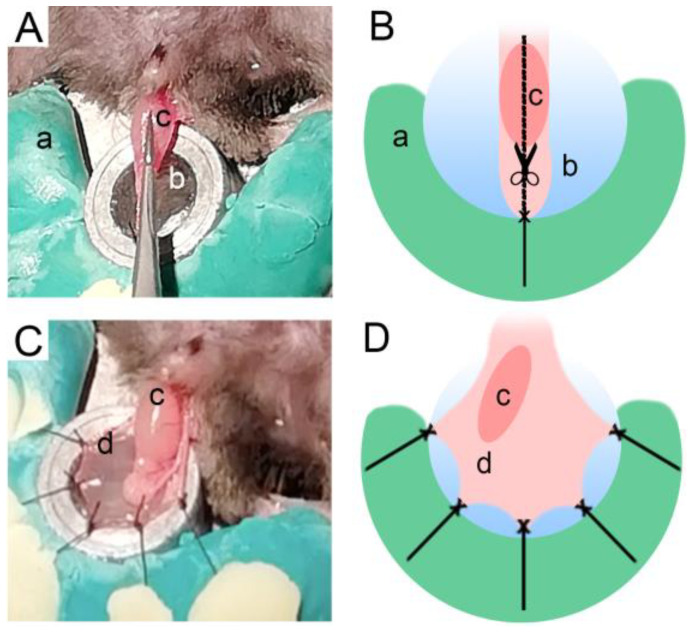
Cremaster preparation. Photographed (**A**,**C**) and illustrated (**B**,**D**) representations of the cremaster preparation procedure. (a) Plasticine; (b) lamella; (c) testicle; (d) cremaster.

Before beginning the cremaster exposure and dissection, the fur had to be removed from the pelvic region of the mouse. Next, the scrotum was extended, and a longitudinal incision was made in the skin. With the testicle and cremaster exposed, a silk suture (B.Braun; Black Silk 5/0 HR20 1/2 45CM) stich made in the cremaster secured it in a stretched position and enabled a longitudinal cut (Figure 2A,B). Finally, the cremaster was spread over the lamella using stiches (Figure 2C,D).

The support was then mounted on a confocal microscope (Leica SP8 MP, Leica Microsystems, Wetzlar, Germany) with a 25× water-immersion objective lens (L25×/0.95 W; Leica PL IRAPO; Leica Microsystems, Wetzlar, Germany). Thus, it was possible to superfuse the tissues with NaCl 0.9% warmed at 37 °C. The saline superfusion was chosen because preliminary studies (data not shown) demonstrated that during a short period of time (time of acquisition), the use of a saline solution does not affect the leukocyte dynamics. This microscope also accommodates a camera (JAI CV-S3200; Infaimon, Portugal), which enabled the recording and post-analysis of the resulting images. A blue fluorescence lamp (Leica EL6000; Leica Microsystems, Wetzlar, Germany) without filters, also connected to the microscope, was used to visualize the EGFP marked neutrophils when analyzing the ear lobe. On the other hand, a 488 nm laser was used to visualize the neutrophils when performing this assay on the cremaster (confocal microscopy).

For each mouse and tissue, three areas of each tissue (cremaster and ear lobe) were randomly selected and recorded for five minutes, and the leukocytes’ dynamics were measured per minute, in preferably straight vessels. For the results’ analysis, it was established that an adhering leukocyte had to remain stationary for 60 s or longer, and quantified in a selected distance of 100 μm. On the other hand, if the leukocytes moved slower than the flux of erythrocytes through a pre-set distance of 100 μm, they were considered rolling leukocytes. The velocity of the rolling leukocytes (μm/s) was manually calculated using a timer to measure the time it took an aleatory leukocyte to flow through 100 μm of 3 vessels per animal.

### 2.5. Determination of Biomarkers Concentrations in the Plasma

Blood was collected from the animals under anesthesia by cardiac puncture using a heparinized tube and syringe and centrifuged for 5 min at 11,000× *g* to separate the plasma. The plasma was then stored at −20 °C for further analysis. Subsequently, the levels of IL-1β in the plasma of the mice were determined using the enzyme-linked immunosorbent assay (ELISA), according to the instructions of the kit (RAB0274, Sigma-Aldrich, St. Louis, MO, USA). Finally, the mice were euthanized with an injection of sodium pentobarbital (Eutasil, 200 mg/mL; Ceva, Portugal) 120 mg/kg.

### 2.6. Statistical Analysis

The data of this work are presented as the mean ± SD (standard deviation) for each group of animals. According to the results obtained with the Shapiro–Wilk test, all the data presented in this study, except the number of adherent leukocytes and leukocytes rolling velocity in the ear lobe of the control groups, were normally distributed. Therefore, the difference between groups was analyzed using the unpaired *t*-test. Values of *p <* 0.05 were considered to represent statistically significant differences. All statistical analysis was calculated with the Prism Software (GraphPad Prism^®^, version 8, San Diego, CA, USA).

## 3. Results

**Figure 3:**
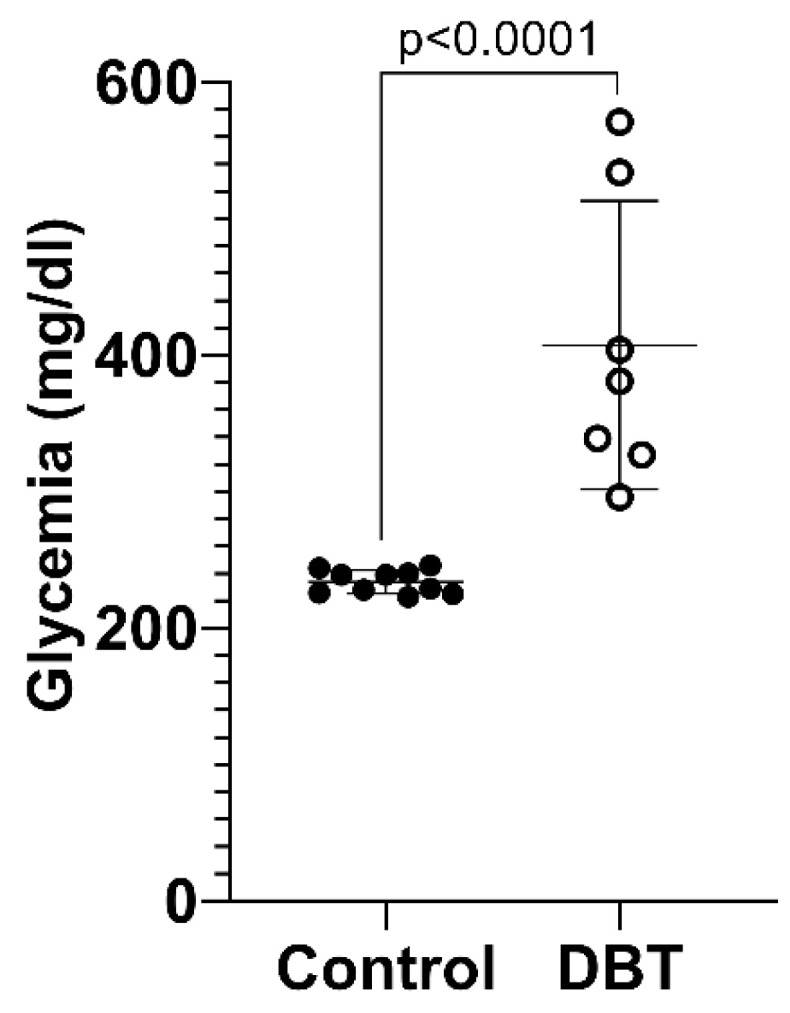
Glycemia monitorization. Mean values (±standard deviation) of glycemia (mg/dL) for non-diabetic (control) and diabetic (DBT) mice, measured after a period of 4 months. Each point in the graph represents a mouse and its measurements average value. The difference between the glycemia values is statistically significant (*p* < 0.0001).

The mean glycemia for all the mice in each group, measured over the period of 4 months, was 233.9 mg/dL ± 8.543 in the non-diabetic (control) group and 407.4 mg/dL ± 105.7 in the diabetic (DBT) group (Figure 3). The difference between the glycemia values of the control and DBT groups was statistically significant (*p* < 0.0001). All the mice were included in the treatment of these data.

### 3.1. Number of Leukocytes in the Ear Lobe and Cremaster

**Figure 4:**
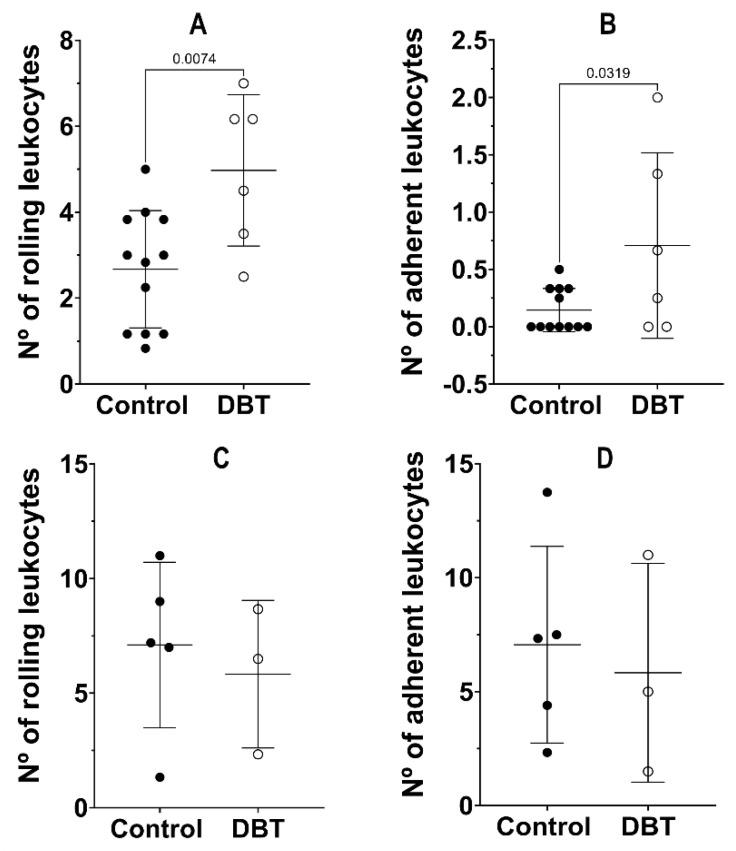
Leukocyte quantification. Mean values (±standard deviation) of the number of rolling (**A**,**C**) and adherent (**B**,**D**) leukocytes in the ear lobe (**A**,**B**) and cremaster (**C**,**D**) muscle for non-diabetic (control) and diabetic (DBT) mice, after a period of 4 months. Each point in the graph represents a mouse and the average value of all measurements. The difference between the number of rolling leukocytes is statistically significant in the ear lobe (*p* = 0.0074) and not statistically significant in the cremaster muscle. The difference between the number of adherent leukocytes is statistically significant in the ear lobe (*p* = 0.0319) and not statistically significant in the cremaster muscle. The number of rolling and adherent leukocytes is significantly higher in the cremaster muscle.

In the non-diabetic animals, the average number of rolling leukocytes in the ear lobe was 2.352 ± 1.534 (Figure 4A), while on the cremaster, it was 7.107 ± 3.609 (Figure 4C). In the diabetic animals, the average number of rolling leukocytes in the ear lobe was 6.014 ± 2.902 (Figure 4A), while in the cremaster, it was 5.833 ± 3.219 (Figure 4C). In the non-diabetic animals, the average number of adherent leukocytes in the ear lobe was 5.797 ± 5.077 (Figure 4B), while on the cremaster, it was 0.1389 ± 0.1667 (Figure 4D). In the diabetic animals, the average number of adherent leukocytes in the ear lobe was 5.833 ± 4.805 (Figure 4B), while in the cremaster, it was 0.6250 ± 0.6620 (Figure 4D).

Equipment failure, death after anesthesia administration, compromised blood flux, and general aspect of the animal are the motives for the exclusion of mice from the analysis. In the ear lobe, the number of rolling leukocytes was significantly higher in the diabetic animals compared to the non-diabetic animals (*p* = 0.0068). On the other hand, in the cremaster, no significant differences were observed between the number of rolling leukocytes of the non-diabetic and diabetic animals (*p* = 0.6345). A similar behavior was observed in the number of adherent leukocytes. While the difference between the number of adherent leukocytes in the control and diabetic groups was not statistically significant, the *p*-value was relatively close to 0.05 (*p* = 0.0518). Thus, it is possible to observe that the number of adherent leukocytes was considerably higher in the diabetic group. In the cremaster muscle, no significant differences were observed between the number of adherent leukocytes in the non-diabetic and diabetic animals (*p* = 0.9923).

### 3.2. Rolling Velocity of Leukocytes in the Ear Lobe and Cremaster

**Figure 5:**
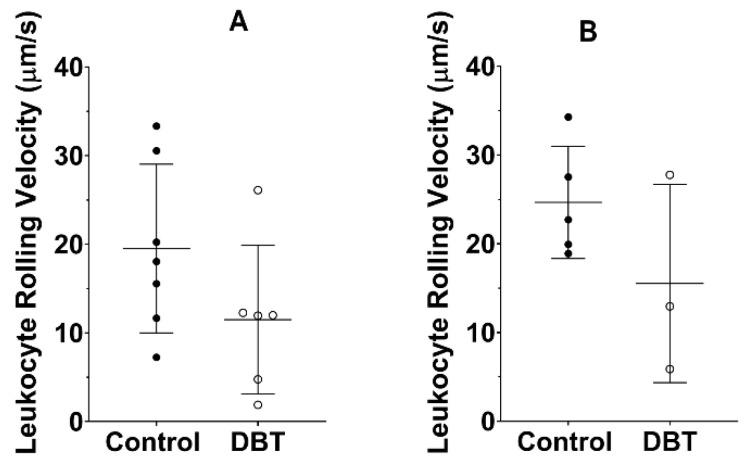
Leukocytes’ velocity. Mean velocity values (±standard deviation) in the ear lobe (**A**) and cremaster muscle (**B**) for non-diabetic and DBT mice after a period of 4 months. Each point in the graph represents a mouse and its measurements average value. The differences between the groups are not statistically significant in both areas (cremaster muscle and ear lobe).

In the ear lobe (Figure 5A), the average rolling velocity of the leukocytes of non-diabetic animals was 19.52 μm/s ± 9.514, while in the diabetic animals, it was 11.50 μm/s ± 8.391. Following a similar behavior, in the cremaster (Figure 5B), the average rolling velocity of the leukocytes of non-diabetic animals was 24.67 μm/s ± 6.328, while in the diabetic animals, it was 15.54 μm/s ± 11.17. In both tissues (ear lobe and cremaster muscle), no statistically significant differences were obtained. However, it was possible to observe through the average values that the rolling velocity of the leukocytes appeared to be generally higher in the non-diabetic mice.

### 3.3. IL-1β Quantification in Plasma

**Figure 6:**
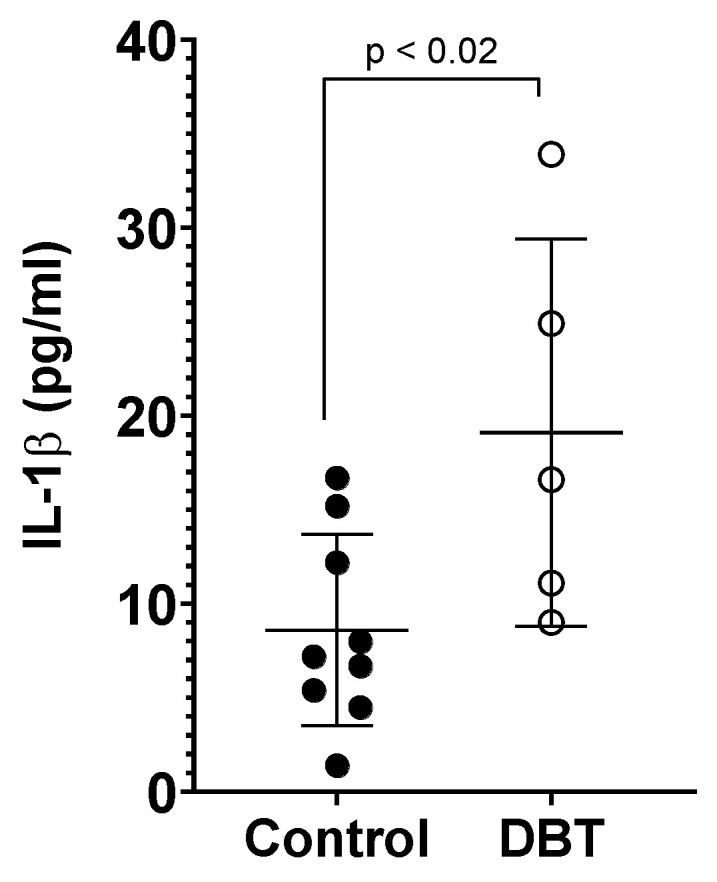
IL-1β plasmatic concentration. Mean values (±standard deviation) of plasmatic concentration of the IL-1β cytokine for non-diabetic and DBT mice after a period of 4 months. Each point in the graph represents a mouse and its measurements average value. The difference between the groups was statistically significant (*p* = 0.0234). The plasmatic concentration of IL-1β in the diabetic mice was significantly higher.

In the non-diabetic animals, the average IL-1β plasmatic concentration was 9.000 pg/mL ± 5.821 (Figure 6), while in diabetic animals, it was 19.10 pg/mL ± 10.31 (Figure 6). Besides the motives that were previously discussed, in this experiment, mice were also not included due to insufficient volumes of collected plasma. The IL-1β plasmatic concentration was significantly higher in diabetic animals, compared to the non-diabetic animals.

## 4. Discussion

According to the glycemia monitorization values, 4 months was sufficient for the mice treated with STZ to develop severe diabetes, because the average glycemia value for the diabetic group was above 400 mg/dL and it had a statistically significant difference compared to the non-diabetic group (glycemia < 250 mg/dL) [2,28]. In the present study, we used young animals (mice developed diabetes for 4 months), and thus their ear lobes were thinner. We started by assessing if 4 months was enough to develop inflammation to a stage that would have an impact on microcirculation. The ear lobe reported a significant increase in the number of rolling leukocytes and the number of adherent leukocytes in the diabetic mice, compared to the non-diabetic mice. Accordingly, considering the differences between the average values, although not statistically significant, the velocity of the rolling leukocytes also appeared to have a trend of decrease in the diabetic animals, which related to the increased number of adherent leukocytes. The significant increase in the plasmatic IL-1β concentration in the diabetic mice indicated that the diabetic mice developed chronic systemic inflammation, which was also reported by the results of the intravital microscopy collected in the ear lobe. On the other hand, in the cremaster muscle, considering the number of rolling leukocytes and the number of adherent leukocytes, the comparison between the diabetic and non-diabetic mice did not reveal any significant differences. However, even though the difference was not statistically significant, the average values of the leukocytes’ rolling velocity appeared to have a trend to decrease in the cremaster of diabetic mice, as was observed in the ear lobe. The similarity between the results of the studied parameters in the cremaster of the diabetic mice and the non-diabetic mice might be explained by the local trauma caused by the surgery, which leads to alterations in the hemodynamics of the microvasculature, with consequent increase in leukocytes trafficking [21,22,29]. Another aspect that has to be considered is that the experiment cannot be taken into account when blood flux is completely compromised. Since that happened approximately four out of nine times, mostly in the diabetic group, this resulted in smaller groups in the cremaster assays than in the ear lobe assays, which made it difficult and, eventually, hampered the comparison between the diabetic and non-diabetic mice. Overall, it is possible to conclude that in the experimental conditions used, namely, the inflammation development period and mice’s age, the intravital microscopy technique can be successfully performed using the ear lobe. This tissue shows clear and great advantages compared to the cremaster muscle. Cremaster surgery introduces more variability to the experiment, which is a consequence of the degree of trauma of the tissue, which in turn is largely dependent on idiosyncratic personal characteristics of the researcher conducting the experiments. Cremaster trauma biases the hemodynamics of the microvasculature, with consequent frequent discard of animals. Adding to the fact that only male mice can be used, this represents more time and resources spent on an experiment. Other authors have already used other less invasive IVM techniques such as ocular IVM [30]. Compared with the cremaster muscle, this is a less invasive technique, although the injection of compounds for, e.g., an inflammatory screening needs to be performed intravitreally, which is a difficult and not so simple procedure. In sum, the ear lobe preparation for the IVM technique is much quicker, non-invasive, and simpler. Thus, it is possible to save time in each experimental procedure, and the period for the researcher to perfect the technique is shorter. Moreover, the gender dimension in the research plans can be fully explored, which will result in less animals discarded. Finally, it is important to stress that the use of the ear lobe, instead of the cremaster, can also be very advantageous in experiments where the aim is to apply the IVM through time in the same animal. This is impossible using the cremaster, because the animals must be sacrificed after a single batch of recordings.

## 5. Conclusions

This study allowed the observation and validation of the effect of systemic chronic inflammation in the microcirculation through the application of IVM in the ear lobe. It was demonstrated that the ear lobe can be used to quantitatively assess the inflammatory status of animals through the observation and recording of the microvasculature of the ear lobe (Figures S1 and S2). Monitoring microcirculation is important in many fields since alterations in microcirculatory blood flow have been identified in several disease processes [31]. A non-invasive methodology opens new possibilities for the use and application of this technique [32].

Moreover, the results obtained through the application of the IVM to the cremaster were not conclusive, revealing intrinsic limitations of this assay that stem from its invasiveness. This work brings new insights that will contribute to a more reliable, cost-effective, and timesaving use of IVM, even to studies in which the gender dimension needs to be accounted for and/or longitudinal follow-up over time is required.

## Data Availability

The data presented in this study are available on request from the corresponding author.

## Supplementary Materials

The following supporting information can be downloaded at: https://www.mdpi.com/article/10.3390/life13040887/s1, Figure S1: Cremaster muscle microcirculation in control (non-diabetic) mice. In green, leukocytes can be seen rolling along the vascular wall. Figure S2: Ear lobe microcirculation in control (non-diabetic) mice. Leukocytes rolling along the vascular wall can be seen in green.

## Abbreviations

Intravital microscopy	IVM
Diabetes mellitus	DM
Streptozotocin	STZ
Postcapillary venules	PCV
